# Supplementary material for: TR3-56 and Treg Regulatory T Cell Subsets as Potential Indicators of Graft Tolerance Control in Kidney Transplant Recipients
Source: Int J Mol Sci. 2024 Oct 2;25(19):10610. doi: 10.3390/ijms251910610 (PMC11477056; doi:10.3390/ijms251910610)
Supplement: Supplementary file 1 [file ijms-25-10610-s001.zip › ijms-3164734-supplementary.pdf]

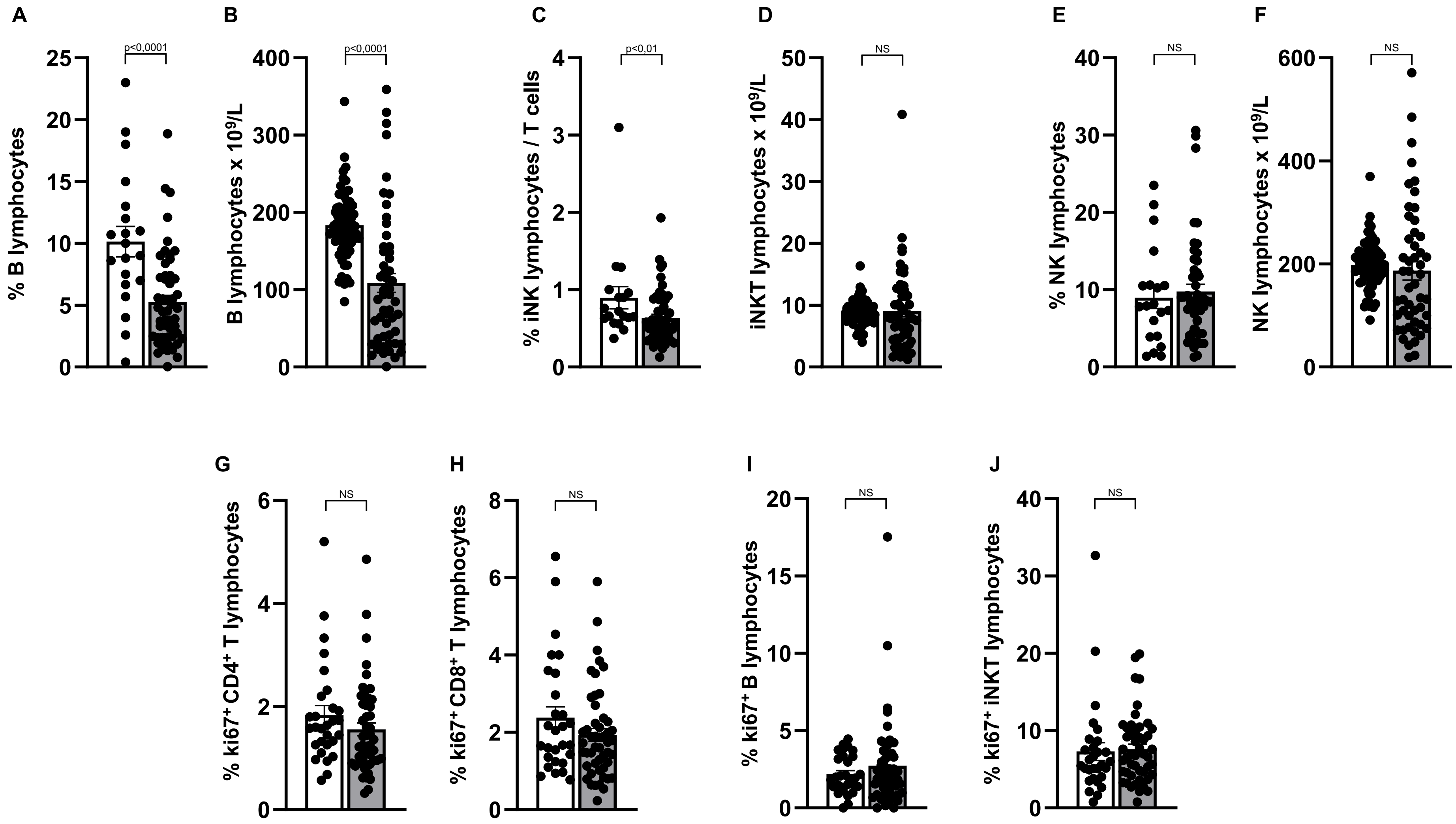


Supplementary Figure S1

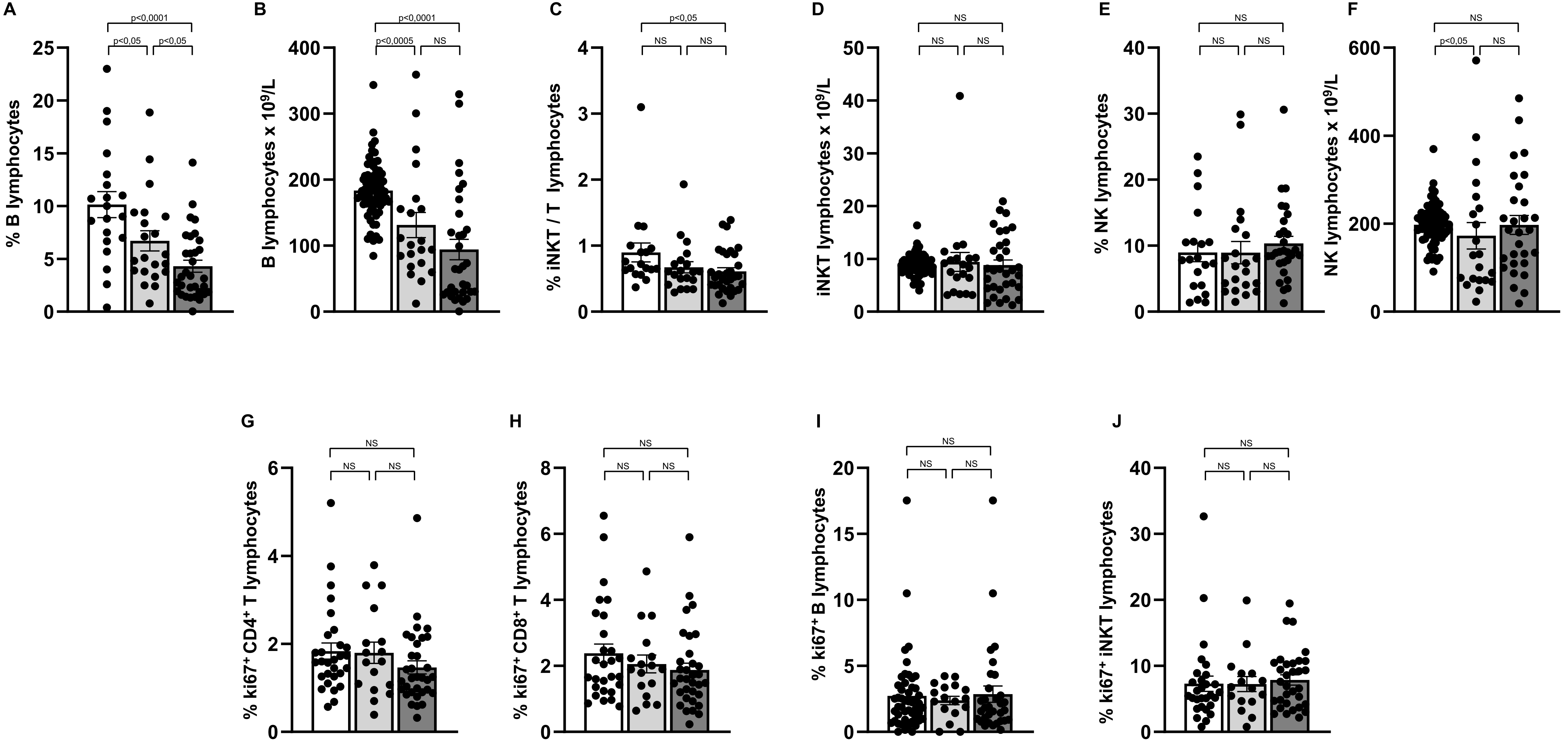


Supplementary Figure S2

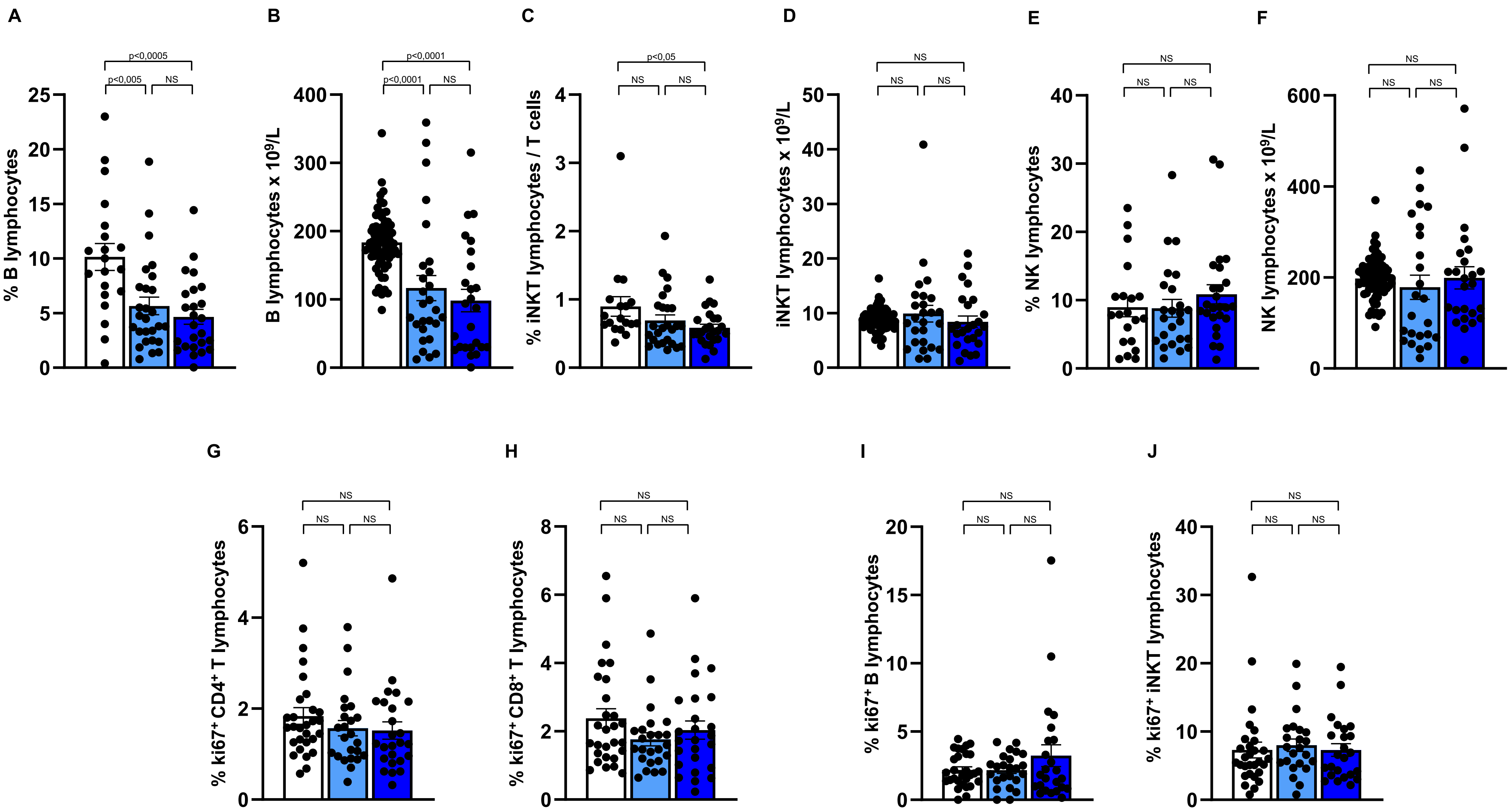


Supplementary Figure S3

## Legends to supplementary figures:

**Supplementary Figure S1. Reduced amount of the circulating iNKT and B lymphocytes characterise a cohort of allograft kidney recipients showing no rejection episodes, no infections and no changes in immuno-suppression therapy in the previous six months.** White and grey columns indicate data obtained in healthy controls and kidney transplanted subjects, respectively. (**a, b**) Indicate percentage and number of circulating B cells, as indicated; (**c, d**) Indicate percentage and number of the circulating iNKT subset; (**e, f**) Indicate percentage and number of the circulating NK cells, respectively; (**g, h, I, j**) Refer growth ability, as represented by intracellular expression of the ki67 molecule, of the circulating $CD4^+$, $CD8^+$, iNKT T cells and of B lymphocytes, respectively; Statistical evaluation of data has been performed by means of the Mann-Whitney test. Statistical significance values are indicated.

**Supplementary Figure S2. Allograft kidney recipients with highest level of circulating $T_{R3\text{-}56}$ regulatory T cells show significant decreased percentage of circulating B lymphocytes when compared with the counterpart.** White columns indicate healthy controls; light and dark grey columns indicate transplanted subjects showing circulating $T_{R3\text{-}56}$ levels <9.16% or ≥ 9.16% of the T cell population, respectively; the 9.16 cut-off value has been obtained by increasing of three standard errors the median value observed in healthy controls (See patient and method section for details). (**a-f**) Indicate percentage and number of the circulating B, iNKT and NK lymphocytes, as indicated; (**g-j**) Show comparative analysis of growth ability of $CD4^+$, $CD8^+$, iNKT and B lymphocytes, between healthy controls and kidney transplant recipients showing $T_{R3\text{-}56}$ levels <9.16% or ≥ 9.16% of the T cell population, respectively; Statistical evaluation of data has been performed by means of the Mann-Whitney test. Statistical significance values are indicated.

**Supplementary Figure S3. Analysis of the immune profile of kidney transplant recipients classified according to the Stable or Unstable disease status.** White columns indicate healthy controls; light and dark blue columns indicate transplanted subjects categorised, according to their clinical and laboratory profile, as belonging to the Stable or Unstable transplant recipient sub-group, respectively. See Patient and Methods section for details. (**a-f**) Indicate percentage and number of circulating B, iNKT and NK lymphocytes; (**g-j**) Indicate growth ability, as represented by intracellular ki67 expression, of $CD4^+$, $CD8^+$, iNKT T cells and B lymphocytes, respectively; Statistical evaluation of data has been performed by means of the Mann-Whitney test. Statistical significance values are indicated.
